# Deafness DFNB128 Associated with a Recessive Variant of Human *MAP3K1* Recapitulates Hearing Loss of *Map3k1*-Deficient Mice

**DOI:** 10.3390/genes15070845

**Published:** 2024-06-27

**Authors:** Rabia Faridi, Rizwan Yousaf, Sayaka Inagaki, Rafal Olszewski, Shoujun Gu, Robert J. Morell, Elizabeth Wilson, Ying Xia, Tanveer Ahmed Qaiser, Muhammad Rashid, Cristina Fenollar-Ferrer, Michael Hoa, Sheikh Riazuddin, Thomas B. Friedman

**Affiliations:** 1 Laboratory of Molecular Genetics, National Institute on Deafness and Other Communication Disorders (NIDCD), National Institutes of Health (NIH), Bethesda, MD 20892, USA; rabia.faridi@nih.gov (R.F.); rizwanyousaf@gmail.com (R.Y.); sayaka.inagaki@nih.gov (S.I.); wilsone2@nidcd.nih.gov (E.W.); cristina.fenollarferrer@nih.gov (C.F.-F.); 2 Auditory Development and Restoration Program, National Institute on Deafness and Other Communication Disorders (NIDCD), National Institutes of Health (NIH), Bethesda, MD 20892, USA; rafal.olszewski@nih.gov (R.O.); shoujun.gu@nih.gov (S.G.); michael.hoa@nih.gov (M.H.); 3 Genomics and Computational Biology Core, National Institute on Deafness and Other Communication Disorders (NIDCD), National Institutes of Health (NIH), Bethesda, MD 20892, USA; morellr@nidcd.nih.gov; 4 Department of Environmental Health, College of Medicine, University of Cincinnati, Cincinnati, OH 45267, USA; xiay@ucmail.uc.edu; 5 Department of Molecular Biology, Shaheed Zulfiqar Ali Bhutto Medical University, Sector G-8/3, Ravi Road, Islamabad 44000, Pakistan; tanveeraq@szabmu.edu.pk; 6 Department of Biotechnology, Institute of Biochemistry, Biotechnology and Bioinformatics, The Islamia University of Bahawalpur, Bahawalpur 63100, Pakistan; m.rashidmukhtar@yahoo.com; 7 Allama Iqbal Medical Research Center, Jinnah Burn and Reconstructive Surgery Center, University of Health Sciences, Lahore 54550, Pakistan; riazuddin@aimrc.org

**Keywords:** *MAP3K1*, *DFNB128*, SNP genotyping, RNA-Seq, locus heterogeneity

## Abstract

Deafness in vertebrates is associated with variants of hundreds of genes. Yet, many mutant genes causing rare forms of deafness remain to be discovered. A consanguineous Pakistani family segregating nonsyndromic deafness in two sibships were studied using microarrays and exome sequencing. A 1.2 Mb locus (DFNB128) on chromosome 5q11.2 encompassing six genes was identified. In one of the two sibships of this family, a novel homozygous recessive variant NM_005921.2:c.4460G>A p.(Arg1487His) in the kinase domain of *MAP3K1* co-segregated with nonsyndromic deafness. There are two previously reported *Map3k1*-kinase-deficient mouse models that are associated with recessively inherited syndromic deafness. *MAP3K1* phosphorylates serine and threonine and functions in a signaling pathway where pathogenic variants of *HGF*, *MET*, and *GAB1* were previously reported to be associated with human deafness *DFNB39*, *DFNB97*, and *DFNB26*, respectively. Our single-cell transcriptome data of mouse cochlea mRNA show expression of *Map3k1* and its signaling partners in several inner ear cell types suggesting a requirement of wild-type *MAP3K1* for normal hearing. In contrast to dominant variants of *MAP3K1* associated with Disorders of Sex Development 46,XY sex-reversal, our computational modeling of the recessive substitution p.(Arg1487His) predicts a subtle structural alteration in *MAP3K1*, consistent with the limited phenotype of nonsyndromic deafness.

## 1. Introduction

**Figure 1:**
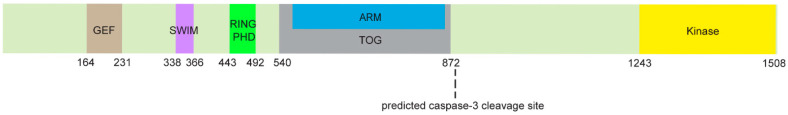
Protein structure of human *MAP3K1* modified from [17]. GEF, putative Guanine Exchange Factor domain; SWIM, SWI2/SNF2 and MuDR domain; RING/PHD, RING-CH-C4HC3_ZSWM2 with Plant Homeodomain motif; TOG, tumor overexpressed gene; ARM, armadillo repeats; Kinase, kinase domain. Reported pathogenic variants of *MAP3K1* and their associated phenotypes are listed in Table S3. The light green regions are not recognized as amino acid sequences belonging to reported domains.

In the auditory systems of diverse animals, the mechano-electrical transduction of sound involves strikingly similar cytoarchitectures. For mice, genetic screens for hearing loss (HL) have identified a variety of mutant genes that are candidates for yet-to-be-discovered inherited human deafness lacking a molecular diagnosis [1,2,3,4]. One example is two different recessive variants of mouse *Map3k1* associated with syndromic deafness [5,6]. *MAP3K1* (mitogen-activated protein kinase kinase kinase one, EC 2.7.11.25, also known as MEKK1, MEKK, MAPKKK1) is one of at least 538 human genes encoding kinases [7,8,9,10]. *MAP3K1* is a large protein of 196 kD with multiple functional domains and has diverse roles in numerous signaling cascades [11,12,13,14]. Its kinase domain phosphorylates serine and threonine of the MAP2Ks, which in turn activate the MAPKs to prevent apoptosis [7,10]. However, when mouse *MAP3K1* is cleaved by caspase-3 at residues DEVD from 871 to 874 (UniProt P53349), the C-terminal fragment becomes pro-apoptotic. In the case of human *MAP3K1*, there is a predicted caspase-3 cleavage site at residues DTLD from 875 to 888 (UniProt Q13233) [13,15,16]. The RING/PHD domain of *MAP3K1* has E3 Ubiquitin ligase activity (Figure 1) that can decrease ERK1/2 activity [12].

The human *MAP3K1* gene is located on 5q11.2 and has 20 exons encoding a 1512-amino-acid residue protein. Dominant missense variants of human *MAP3K1* cause Disorders of Sex Development (DSDs, OMIM 613762, SRXY6), a sex-limited 46,XY gonadal dysgenesis phenotype with a partial or complete sex reversal to a female phenotype [17,18,19,20]. Nearly all the DSD-associated *MAP3K1* variants are clustered in the N-terminal Guanine Exchange Factor (GEF), SWI2/SNF2 and MuDR (SWIM), RING finger including a PHD motif (RING/PHD), and armadillo repeats (ARM)/tumor overexpressed gene (TOG) domains (Figure S4 and Table S3). One variant, p.(Ala1443Val), associated with DSDs is located in the kinase domain of *MAP3K1* [21]. Some *MAP3K1* DSD-associated variants show altered substrate binding and increased phosphorylation of its substrates such as p38 and ERK1/2 [17]. HL was unlikely to be missed in the published individuals with DSDs in particular where the senior author in another study has investigated human deafness [22]. Moreover, HL has not been previously reported as part of the DSD phenotype in humans [17]. 

Homozygosity for two different mouse *Map3k1* variants results in profound deafness by nine weeks after birth [5,6]. The *Map3k1^tm1Yxia^* allele (also known as *Mekk1*^−^ [23]; *Mekk1*^lacz^ [24]; *Mekk1*^∆KD^) is a gene trap in which 1188 amino acid residues of the *MAP3K1* N-terminal domain are fused with a β-galactosidase reporter (LacZ) replacing the entire C-terminal kinase catalytic sequence [23,24], resulting in the expression of *MAP3K1*-β-galactosidase fusion protein. A second *Map3k1* mutant allele (goya mouse, *Map3k1^goya^*) arose in an ENU-mutagenesis screen for HL. A single-nucleotide variant was identified in *Map3k1* at a splice donor site (IVS13+2T>C). RT-PCR analysis of P1 *Map3k1* goya inner ear cDNA revealed no wild-type transcripts and two aberrant transcripts, one of which deletes 27 inframe amino acid residues which does not change the amino acid sequence of the kinase domain of *MAP3K1*. The second *Map3k1* goya transcript completely excludes the sequence of exon 13, resulting in a downstream premature translation stop codon. If translated, the protein would lack the C-terminal 770 residues of *MAP3K1* which includes the kinase domain [5]. In addition to profound deafness, *Map3k1^tm1Yxia/tm1Yxia^* and *Map3k1^goya/goya^* have an “eye-open at birth” phenotype [24], retinal degeneration [25], and insufficiencies of the immune system and wound-healing [26,27]. 

*MAP3K1* is expressed in many cell types and has multiple necessary functions throughout the body [26]. In the mouse inner ear, the stria vascularis generates a positive 80-millivolt potential in the endolymph, a potassium-rich fluid that bathes the apical surface of the organ of Corti [28]. Single-cell RNA-Seq (sc-RNA-Seq) data for *Map3k1* in the mouse auditory system suggest expression in the reticular lamina created by Deiters’ and pillar cells, Claudius cells, stria vascularis, and tympanic border cells as well as Reissner’s membrane. One or more of these cell types in the inner ear may require wild-type *MAP3K1* function for normal hearing. Echoing the mouse auditory phenotype associated with *Map3k1*-kinase-deficient alleles, here, we report a human family segregating a novel recessive variant c.4460G>A p.(Arg1487His) in *MAP3K1* associated with nonsyndromic severe-to-profound deafness, suggesting that this variant results in a focused phenotype that damages the auditory system. 

## 2. Materials and Methods

Family PKDF1419 was ascertained in Pakistan.

### 2.1. Informed Consent and Institutional Review Board (IRB) Approvals 

**Figure 2:**
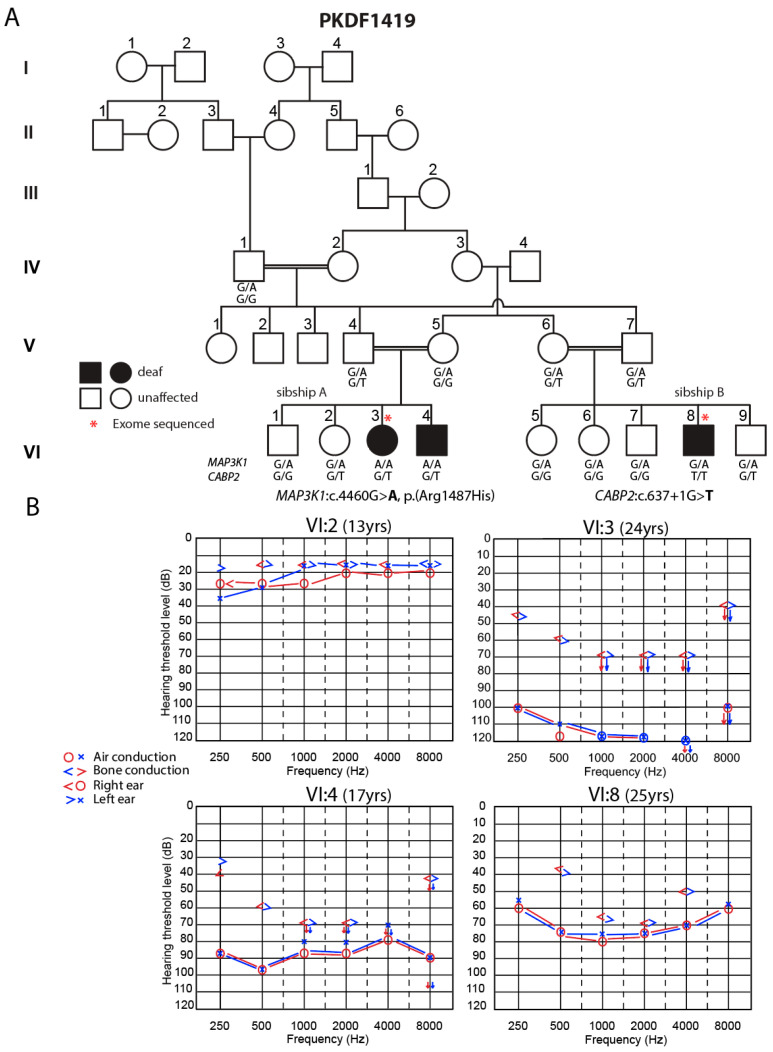
Hearing loss associated with biallelic variants of human *MAP3K1*. (**A**) Pedigree of a six-generation family with genotypic data from fourteen individuals. Circles and squares illustrate female and male individuals, respectively. Filled circles and squares refer to deaf individuals, open circles and squares represent individuals with normal hearing thresholds, and * indicates individuals with exome sequencing data. For *MAP3K1*, G is the wild-type allele, while A is the mutant allele. For CABP2, G is the wild-type allele, and T is the mutant allele. (**B**) Audiograms of individual VI:2, VI:3, VI:4, and VI:8. Individual VI:2 shows hearing thresholds within normal range. VI:3 has bilateral profound SNHL, and VI:4 has bilateral, severe-to-profound SNHL, whereas individual VI:8 has a moderate-to-severe degree of HL. Arrows indicate no response to the auditory stimulus at the indicated levels.

All participants provided written informed consent after Institutional Review Board (IRB) approvals from the National Centre of Excellence in Molecular Biology, University of the Punjab (FWA00017939), and from the Combined National Institutes of Health (NIH) IRB (protocol OH93DC0016). Signed informed consent was obtained from fourteen individuals of Pakistani Family PKDF1419 to study hereditary HL segregating as an autosomal recessive trait (Figure 2A).

### 2.2. Audiology Testing

Hearing was evaluated by pure-tone (air conduction) audiometry at octave frequencies from 250 to 8000 Hz. Individual VI:2 shows hearing thresholds within normal range, VI:3 has bilateral profound sensorineural HL (SNHL), and VI:4 has bilateral, severe-to-profound SNHL, whereas individual VI:8 has a moderate-to-severe degree of HL. The ages at the time of audiological examination are shown on the audiograms. Arrows indicate no response to the auditory stimulus at the indicated levels. Symbols “o” and “x” denote air conduction pure-tone thresholds at different frequencies in the right and left ear, respectively.

### 2.3. Chromosomal Microarray Single-Nucleotide Polymorphism Genotyping

To identify chromosomal intervals for a homozygous variant associated with deafness segregating in Family PKDF1419, genotyping was performed on genomic DNA (gDNA) samples from two affected and three unaffected individuals (VI:3, VI:4, VI:2, V:4, and V:5) from the family (Figure 2A). Infinium OmniExpressExome-8 v1.4 BeadChips with an Infinium HD Super-assay (Illumina, San Diego, CA, USA) were used to analyze 960,919 single-nucleotide polymorphisms (SNPs) following the manufacturer’s protocol. Illumina GenomeStudio software (v2) was used to evaluate the genotyping data, which had at least a 98% call rate. SNP data were exported from GenomeStudio for further downstream analyses to AutoSNPa (v3) following a published protocol [29].

### 2.4. Exome Sequencing (ES)

gDNA was extracted from peripheral blood leukocytes [30]. A gDNA sample from one affected individual of each branch of the family was initially screened by di-deoxy sequencing using BigDye (Applied Biosystems, Waltham, MA, USA) for pathogenic variants of *GJB2* (*DFNB1A*, OMIM 220290) and *HGF* (*DFNB39*, OMIM 608265). In Pakistan, mutant alleles of these two genes are common causes of deafness [31]. ES was performed with gDNA from two individuals (one from each sibship) of Family PKDF1419 (Figure 2A). Exome libraries were prepared using a Nextera Rapid Capture Exome kit and sequenced using a HiSeq 1500 instrument (Illumina). Computational analyses used the GATK pipeline (Genome Analysis Toolkit, Broad Institute, Cambridge MA, USA) [32] followed by variant calls that were annotated with Annovar v2014_07_14 [33]. Variants were prioritized based on filtering the data with Clinical Insight software (release: 8.1.20220121, Qiagen, Hilden, Germany). Prioritization criteria included variants with a combined annotation-dependent depletion (CADD) score greater than 15 and an allele frequency of less than 0.5% (gnomAD, NHLBI Exome Sequencing and 1000 Genomes projects, Bethesda, MD, USA), and the variant is predicted to be deleterious by at least one of the multiple in silico tools [34]. These tools are used to assess the predicted effect of an amino acid substitution on the protein structure or function in the absence of experimental demonstration of its effect. Variants were verified by Sanger di-deoxy sequencing using an 3500XL genetic analyzer (Applied Biosystems).

### 2.5. Genotyping the Map3k1^tm1Yxia^ Mouse

Genotyping of the *Map3k1^tm1Yxia^* mouse utilized two independent PCR reactions, one for the mutant allele producing an approximate 1.6 kb amplimer and one for the wild-type allele producing an approximate 1 kb amplimer. The primer pair used for the mutant allele is MEKK1N.F 5′-GCTGTTGGAATTTCCTGCTG-3′ and lacZbing.R 5′-AAGCGCCATTCGCCATTCAG-3′. The primer pair used to amplify the wild-type allele is MEKK1kd3.F 5′-CCGCCATCCACTCAATGAAGACG-3′ and MEKK1kd5.R 5′-CCAAAGCGAAACAGCCTTACAGAG-3′. The PCR reaction utilized a thermocycler profile having an initial denaturation at 94 °C for 10 min, 94 °C for 30 s, annealing at 62 °C for 30 s, extension at 72 °C for 1 min, which was repeated for 35 cycles with a 0.3 °C decrease in annealing temperature for each cycle, and a final extension at 72 °C for 1 min. Taq 2X Master Mix (New England Biolabs, Ipswich, MA, USA) was used with a 20.0 µL total reaction volume containing 1.0 µL of each of the forward and reverse primers (10 µM), 6.0 µL of water, and 2.0 µL of gDNA template at 25–50 ng/µL. 

### 2.6. Structural Modeling of the Kinase Domain of Human MAP3K1 

The kinase domain of human *MAP3K14* (also known as NF-kappaB) (PDB id: 4G3D) was selected as the template for the structural modeling procedure as it has the highest coverage (residues 308 to 673), the highest sequence identity (31%), and better correspondence between secondary structural elements and was not bound to an inhibitor or mutated. The initial sequence alignment of the kinase domain between human *MAP3K1* and human *MAP3K14* obtained from HHpred was refined in an iterative process, using conservation scores obtained from the Consurf server [35], positioning the most conserved residues towards the core of the protein and removing gaps in secondary structural elements when needed. In addition, the preliminary structural model obtained after each iteration was evaluated with the ProQ2 score [36]. The final alignment obtained after the refinement process was used during the modelling production run, where 2000 modelling iterations were performed with MODELLER [37]. The selected kinase model of human *MAP3K1* had the highest MODELLER probability distribution function (molPDF) and ProQ2 scores with the best stereochemistry using Procheck v.3.5.4 [38]. A similar procedure was used to obtain a structural model for the kinase domain of human *MAP3K1* family proteins. 

### 2.7. In Silico Splicing Evaluation and RNA-Seq 

Potential aberrant splicing of the variant NM_005921.2:c.4460G>A was evaluated using SpliceAI which is a deep learning-based tool to predict variant effects on splicing (https://spliceailookup.broadinstitute.org/, (accessed on 10 June 2024)) [39]. Values of <0.2 have a low probability of causing an abnormal splice. Values of 0.2 to 0.5 have a predicted splice abnormality that is uncertain, and a value of >0.8 predicts that the variant is likely to cause an abnormal splice event. As a negative control, a silent substitution of *MAP3K1* NM_005921.2 c.4461T>C p.(Arg1487Arg) was evaluated. As a positive control, a reported splice-altering variant *LRP2* NM_004525.3:c.7715+3A>T p.(Gln2573LeufsTer11) was evaluated [40].

### 2.8. Single-Nucleus RNA-Seq

Single-nucleus RNA-Seq datasets of the stria vascularis and adult spiral ganglion neurons and a P7 organ of Corti single-cell RNA-Seq dataset were analyzed for the expression of *Map3k1* [41,42,43]. Violin plots of expression among cell types in the stria vascularis, adult spiral ganglion neurons, and P7 organ of Corti cell types were constructed as in [41,44].

### 2.9. EP Measurements

The method we used for EP measurements employed a glass micro-electrode that was inserted into the round window of the mouse inner ear, as previously described in detail [45,46,47]. For the anesthesia of mice, tribromoethanol (Avertin, 15.1 mg/mL, Winthrop Chemical Co., Bridgewater, NJ, USA) was used at 0.35 mg per gm of body weight. Procedures with mice were approved by the National Institute on Deafness and Other Communication Disorders (NIDCD; ASP1379) of the Animal Care and Use Committee.

## 3. Results

There are two affected and two unaffected children of a consanguineous union between normal hearing parents, V:4 and V:5, of Family PKDF1419 sibship A (Figure 2A). In sibship B of Family PKDF1419, there are four unaffected and one deaf child, VI:8, from a consanguineous marriage of two normal hearing parents. Proband VI:3 is a 24-year-old female (Figure 2A). Her audiogram (Figure 2B) shows bilateral, profound SNHL. Male VI:4 is 17 years old. His audiograms show bilateral, severe-to-profound SNHL. VI:8 is a 25-year-old male in sibship B whose audiogram shows mixed HL characterized by diminished sensorineural and conductive sound transduction (Figure 2A,B). We do not know when the childhood HL segregating in Family PKDF1419 occurred. At the hospital discharge of newborns, the hearing status of neonates of Family PKDF1419 was not documented, as newborn hearing screening in Pakistan was not then available. Subsequently, the parents noted that their two babies were unresponsive to noise.

### 3.1. Genotyping and Sequencing

#### 3.1.1. Chromosomal Microarray and Exome Data Analyses

**Figure 3:**
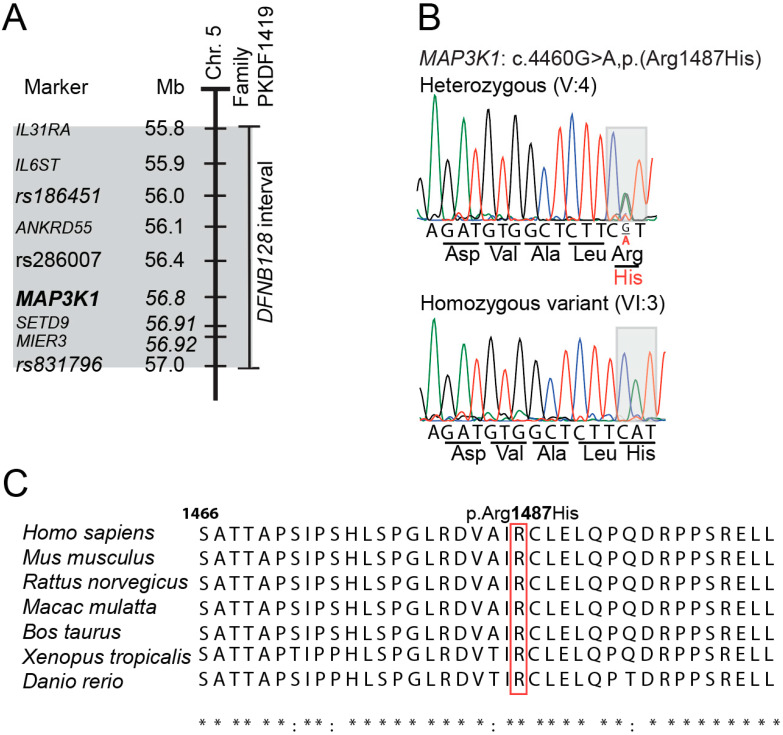
Refinement of the *DFNB128* region of homozygosity (ROH), chromatograms, and sequence alignments. (**A**) The thick vertical bar represents human chromosome 5q. The ROH for deafness segregating in Family PKDF1419 is indicated by a thin vertical bar. The gray shaded region is the DFNB128 ROH with the position of the *MAP3K1* gene in bold. (**B**) Representative chromatograms of the gDNA sequences obtained from individuals V:4 and VI:3 who are heterozygous (G/A) and homozygous (A/A) for the p.(Arg1487His) variant, respectively. The affected codon is shaded in gray. Each color line refers to a base. Green refers to adenine, Red to thymine, Blue to cytosine and Black to guanine. (**C**) Conservation of human Arg1487 residue in *MAP3K1* orthologs shows that Arg1487 residue is conserved among a variety of species. (RefSeq IDs: human; NP_005912.1; mouse NP_036075.1; rat; NP_446339.2; rhesus, XP_002804414.2; cattle, NP_001192835.1; frog, XP_012822348.1; zebrafish; XP_005155564.1.). * indicates identical in all sequences in the alignment; : indicates conserved substitutions have been observed.

With the goal of identifying a single locus of homozygosity associated with the deafness segregating in Family PKDF1419, we genotyped 960,919 SNPs. After analyzing microarray data, one region of SNP homozygosity was not identified for all three affected individuals in the two sibships of consanguineous Family PKDF1419. For sibship A, a shared region of homozygosity on chromosome 5 was observed. After exome sequencing, a predicted pathogenic variant, NM_005921.2:c.4460G>A p.(Arg1487His), of *MAP3K1* was identified within that interval. Sanger sequencing confirmed this recessive variant (Figure 3B). Both hearing parents are carriers of the c.4460G>A allele, as are VI-1 and VI-2, normal hearing siblings of the proband (Figure 2A). Amino acid sequence alignment reveals that the Arg1487 residue is well conserved among vertebrates from humans to frogs and zebrafish (Figure 3C). In the gnomAD database (v4.0.0), the c.4460G>A variant has been observed worldwide three times among 1,613,810 sequenced chromosomes, only once in the South Asian population, indicating the rarity of this variant. The c.4460G>A variant was also not identified in 348 chromosomes from normal hearing ethnically matched control Pakistani gDNA samples that we sequenced. The gDNA sequences of the annotated exons of the six other genes (*MAP3K1*, *SETD9*, *ANKRD55*, *IL31RA*, *IL6ST*, and *MIER3*) located in the 1.2 Mb interval on chromosome 5 were well covered in our exome data, and no convincing biallelic deleterious variants were found.

Analyses of the SNPs using GenomeStudio software (v2.0, Illumina, San Diego, CA, USA) revealed one additional region of homozygosity among two affected individuals of sibship A, which is a 12 Mb region on chromosome 1 (chr1:48.5–60.6 Mb) that includes 74 genes, one of which is *BSND* encoding a chloride channel. Variants of *BSND* are associated with either Bartter syndrome type IV (OMIM 602522) or nonsyndromic deafness *DFNB73* [48]. However, no pathogenic variants were detected in the UTRs and protein coding regions of *BSND*. We cannot rule out the possibility of a homozygous deep intronic variant of *BSND* (Table S1A). 

To date, a second family or a singleton with additional deleterious variants of *MAP3K1* associated with deafness have not been ascertained by us or reported by others. It cannot be ruled out that the c.4460G>A p.(Arg1487His) of *MAP3K1* variant is in linkage disequilibrium with a nearby real causal variant responsible for deafness in sibship A of Family PKDF1419. Yet, two different recessive variants of mouse *Map3k1* associated with deafness are consistent with our supposition that the genetic explanation for nonsyndromic deafness segregating in sibship A of Family PKDF1419 has been identified as c.4460G>A p.(Arg1487His) of *MAP3K1*. 

Locus heterogeneity occurs when variants of two or more different mutant genes are responsible for a similar phenotype segregating among different affected individuals in a single family or in an ethnically or geographically delimited community. Locus heterogeneity for deafness in consanguineous families has been reported previously [31,49]. Family PKDF1419 is another such example of locus heterogeneity. In sibship B (Figure 2A), there are five siblings, one of whom, VI:8, is deaf. ES data for male VI:8 revealed a homozygous wild-type sequence for *MAP3K1*, and thus, the deafness in sibship B is not explained by an altered function of *MAP3K1* as it is in sibship A. However, VI:8 is homozygous for a previously reported splice site variant NM_016366.3:c.637+1G>T p.(Phe164Serfs*4) in the *CABP2* gene encoding Calcium-Binding Protein 2 on chromosome 11. His audiogram indicates a moderate-to-severe degree of HL, as previously described (Figure 2B) [50]. The *CABP2*:c.637+1G>T variant was heterozygous in the two deaf individuals of sibship A, and only their father (V:4) was a carrier of the *CABP2* variant, while their mother (V:5) was homozygous for the wild-type allele of *CABP2* (Figure 2A). The *CABP2*:c.637+1G>T allele was previously identified in three Iranian families segregating *DFNB93* moderate-to-severe HL with this variant on a 0.52 Mb haplotype indicating a single ancestral origin [50]. The c.637+1G>T variant has an allele frequency of 0.0007822 in gnomAD v3.1.2. CABP2 is a modulator of inner hair cell voltage-gated calcium channel Cav1.3 [50,51]. Other recessive variants of *CABP2* have also been reported in families segregating prelingual, moderate-to-severe HL from Pakistan [31], Iran [52], Turkey [53], and Northern Europe [54]. 

#### 3.1.2. In Silico and Computational Homology Modeling Predictions of the Kinase Domain of Human *MAP3K1*

To evaluate the putative impact of the variant NM_005921.2:c.4460G>A on splicing, we performed an in silico analysis including negative and positive controls using SpliceAI (Table S5). Neither an acceptor site loss/gain nor a donor site loss/gain were predicted for NM_005921.2:c.4460G>A, indicating that this variant has no predicted impact on splicing. The dinucleotides GT at 4460 and 4461 of *MAP3K1* are not used as a donor site in the wild type, and the variant of c.4460G>A does not create a new donor or acceptor site. The negative control NM_005921.2:c.4461T>C was predicted to have no impact on splicing. The SpliceAI prediction of the positive control NM_004525.3:c.7715+3A>T showed that the scores of donor loss and donor gain were 0.79 and 0.59, respectively, suggesting the variant alters splicing. In fact, this variant is reported to skip the *LRP2* exon 41 consensus donor splice site, resulting in the retention of 22 bp of intron 41 and a frameshift [40].

**Figure 4:**
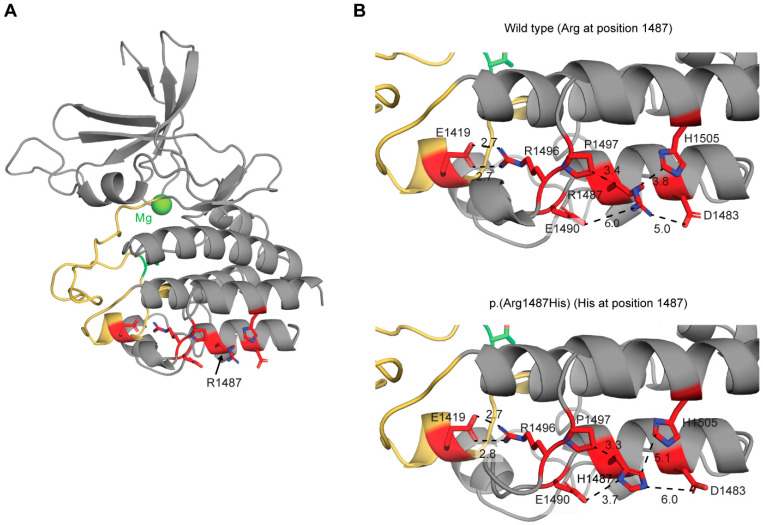
Structural model of the kinase domain of human *MAP3K1* wild type (**A**) and close-up views of residue at position 1487 for the wild type (Arg at the position of 1487) and p.(Arg1487His) variant (His at residue 1487). (**B**) The activating loop of the domain is colored in yellow, while the residues taking part in the interacting network in which Arg1487 participates are colored in red. Blue color indicates nitrogen atom. The magnesium ion is shown as a green sphere in (**A**). Distances between heteroatoms (N, O, C) are shown in Angstroms and represented as dashed lines in (**B**).

The homozygous *MAP3K1*:c.4460G>A variant identified in the two individuals affected in sibship A of Family PKDF1419 has a CADD score of 31, categorizing it as deleterious (Table S2). A CADD score of 20 or higher indicates that a variant is among the 1% most deleterious substitutions [55]. The c.4460G>A variant has a REVEL score of 0.537, categorizing it as having uncertain significance. However, FATHMM-MKL, the likelihood ratio test LRT [56], and MetaRNN [57], a neural network-based ensemble incorporating 16 scores from several in silico programs, predict that c.4460G>A, p.(Arg1487His) is deleterious. The arginine-1487 residue is in the kinase domain at the C-terminus of human *MAP3K1* (residues 1243–1508, RefSeq NP_005912.1) (Figure 4A). To assess how the Arg1487His substitution might influence the structure of the kinase domain, we applied computational homology modeling to predict the structure of the kinase domain of human wild-type *MAP3K1* (arginine at position 1487) and the p.(Arg1487His) variant (histidine at position 1487) using the kinase domain of human *MAP3K14* as a template. The models obtained show a typical bilobed (C-terminal and N-terminal lobes) architecture of protein kinases. The Arg1487 residue is located near the surface of the C-terminal lobe (Figure 4B). Comparison of the wild-type structure and p.(Arg1487His) variant structure shows that the Arg1487His substitution forms weaker hydrogen bonds with neighboring residues, Asp1483, Glu1490, and His1505, with distance differences between heteroatoms of 1.0, 2.3, and 1.3 Å, respectively. The conformation of the activation loop, which is essential for the catalytic activity, appears to be unaffected by these small local structural changes introduced by the Arg1487His substitution (Figure 4B). Glu1419 in the activation loop located close to C-terminal lobe forms two hydrogen bonds with Arg1496. There are no significant changes due to the substitution with histidine at position 1487 (Figure 4B). Taken together, these data suggest that the Arg1487His substitution probably has little impact on kinase catalytic function. However, we stress that the predicted minor but deleterious alteration in the local structure due to the p.(Arg1487His) substitution was expected and is consistent with the limited phenotype of nonsyndromic deafness *DFNB128* in comparison to the severe pleiotropic phenotype of the two reported highly damaging alleles of mouse *Map3k1* [5,6] and with the complex DSD phenotype due to dominant variants of *MAP3K1* [17,18,19,20].

#### 3.1.3. Single-Cell Transcriptome Analysis of *Map3k1* Shows Expression in Distinct Regions of the Cochlea

**Figure 5:**
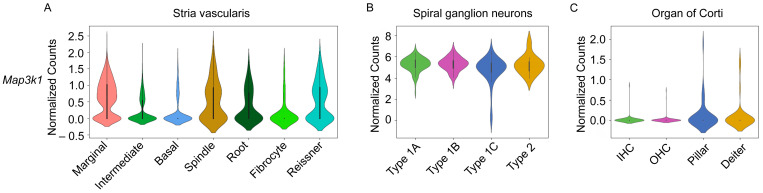
Single-nucleus RNA-Seq data on the stria vascularis extracted from our previous work [41] and single-cell RNA-Seq data on the spiral ganglion neurons (SGNs) [42] and organ of Corti [43] demonstrate the expression of *Map3k1* in regions of the cochlea. (**A**) In the stria vascularis (1st violin plot on left), *Map3k1* is expressed in marginal cells of the stria vascularis, spindle cells, root cells, and Reissner’s membrane from P30 CBA/J mice. (**B**) Amongst SGNs (2nd violin plot in middle), *Map3k1* expression is observed across all SGN subtypes from P25 to 27 of CBA/CaJ mice. (**C**) Minimal expression of *Map3k1* is detected in inner hair cells (IHCs), outer hair cells (OHCs), pillar, or Deiters’ cells in the organ of Corti from P7 CD1 mice (3rd violin plot on right).

The analysis of the single-cell transcriptome (scRNA-Seq) in publicly available datasets and from our single-nucleus RNA-Seq (snRNA-Seq) data from the mouse inner ear demonstrates the expression of *Map3k1* and some of its upstream signaling partners in different cell types of the stria vascularis (SV) and in the spiral ganglion neuron (SGN) regions of the mouse cochlea. In the stria vascularis, *Map3k1*, *Hgf*, and *Gab1* are all expressed in marginal cells [41] (Figure 5A). *Met*, encoding the receptor for HGF, is known to be expressed by SV intermediate cells [44]. The snRNA-Seq expression data for *Hgf* and *Met* have been previously validated by our group using single-molecule fluorescence in situ hybridization (smFISH) in the adult mouse stria vascularis [45]. Interestingly, *Map3k1*, *Hgf*, *Gab1*, *Mettl13*, and *Spry2* are all expressed in spiral ganglion neurons. Except for *Gab1*, which appears to be expressed specifically in type 1C spiral ganglion neurons, the remainder of these genes is expressed across all spiral ganglion neuron cell subtypes [42] (Figure 5B and Figure S2). However, *Map3k1* and its upstream signaling partners appear to be negligibly expressed amongst organ of Corti cell types including inner and outer hair cells and pillar and Deiters’ cells (Figure 5C). 

#### 3.1.4. *Map3k1^tm1Yxia/tm1Yxia^* Mice Have a Wild-Type Endocochlear Potential 

In the inner ear, *Map3k1* is expressed in many different cell types, including Claudius cells, Hensen cells, Reissner’s membrane, Deiters’ cells, and the basilar membrane [5,6,43], and prominently in the stria vascularis. We asked whether the deafness of the *Map3k1^tm1Yxia/tm1Yxia^* mouse is due to loss or diminution of the endocochlear potential (EP), which was not reported for the *Map3k1^goya^* or *Map3k1^tm1Yxia^* mouse models [5,6]. Blind to genotype, EP was measured only in the left ear. The gender distribution for wild-type mice was three females and three males; for heterozygotes, it was three females and two males, and for homozygotes, it was two females and three males. *Map3k1^tm1Yxia^*^/*tm1Yxia*^ mice were obtained from crosses between heterozygous parents. Eight male and eight female mice were tested for EP between P36 and P62. We observed no significant difference from the expected 90–100 millivolts. EPs of the wild type for either heterozygotes or homozygous mutant mice (Figure S1). These data point to elsewhere in the auditory system being a primary cause of deafness in the *Map3k1^tm1Yxia/tm1Yxia^* mouse.

## 4. Discussion

Family PKDF1419 is segregating recessively inherited nonsyndromic deafness. Following homozygosity mapping and exome sequencing, the two sibships of Family PKDF1419 were found to have different molecular genetic diagnoses for their deafness. This phenomenon is referred to as inter-sibship familial locus heterogeneity [49]. In Family PKDF1419, sibship B is segregating a previously reported founder variant of *CABP2* [50], while sibship A is segregating a novel substitution of a highly conserved residue located in the kinase domain of *MAP3K1* that is associated with nonsyndromic deafness *DFNB128*, a newly reported locus for human HL. Several reported dominant variants of human *MAP3K1*, which are predominantly substitutions located in the N-terminal domains of *MAP3K1*, cause DSDs, sex-limited 46,XY gonadal dysgenesis (Tables S3 and S4) [18]. Individuals with *DFNB128* deafness do not show a DSD phenotype. Male individual VI:4 in Family PKDF1419 has SNP probes on the Y chromosome. DSD individuals appear not to have been evaluated for a hearing phenotype. It is likely that significant hearing loss would have been noticed and reported. In addition to the novel recessive variant of *MAP3K1* that we identified in our family reported here, there is a carrier of a likely benign variant of *MAP3K1*, p.(Arg183Gln) (rs1454725137), reported in a deaf individual whose phenotype was explained by a dominant variant of *MYH9* [58].

To date, a second family or a singleton with additional deleterious variants of *MAP3K1* associated with deafness have not been ascertained by us or reported by others. It cannot be ruled out that the c.4460G>A p.(Arg1487His) of *MAP3K1* variant is in linkage disequilibrium with a nearby causal variant responsible for deafness in sibship A of Family PKDF1419. Yet, two different recessive variants of mouse *Map3k1* associated with deafness are consistent with our supposition that the genetic explanation for nonsyndromic deafness segregating in sibship A of Family PKDF1419 has been identified as c.4460G>A p.(Arg1487His) of *MAP3K1*.

*MAP3K1* functions in the Wnt and HGF/MET signaling pathways [59,60]. In the HGF signaling pathway, variants of *HGF* (DFNB39) [61], *MET* (DFNB97) [62], and *GAB1* (DFNB26) [63] are associated with human HL. Similarly, mutant alleles of mouse *Hgf* are associated with deafness [45,61] (Figure S3). A future study of an engineered mouse corresponding to the human *MAP3K1* p.(Arg1487His) variant and a mouse with tandem-tagged endogenous *MAP3K1* coupled with mass-spectroscopy data might reveal the substrates and interacting partner proteins of *MAP3K1* in the inner ear of which there are already 28 reported binding partners [26]. Perhaps additional protein interactors exist in the vertebrate inner ear. A comprehensive study to explore the predicted pathogenicity of the human Arg1487His substitution could be undertaken by engineering a mouse *Map3k1* model with the corresponding Arg1468His variant of this conserved region of *MAP3K1* (Figure 3C). But even without these additional data, we emphasize that two different mutant *Map3k1* mouse models are deaf, supporting the supposition that wild-type *MAP3K1* is necessary for human hearing. 

The inner ear appears to be especially sensitive to hypomorphic variants. We posit that *MAP3K1* falls into this category of a ubiquitously expressed gene where subtle changes result in a focused phenotype, in this case, deafness. Conversely, if one ascertains families segregating severely debilitating syndromic forms of deafness, we predict more deleterious variants will be identified in the very same genes previously thought only to result in, or be limited to, nonsyndromic deafness. In support of our supposition that p.(Arg1487His) is associated with human deafness, (1) we identified a shared 1.2Mb region of homozygosity on chromosome 5q11.2 for the two affected individuals in Family PKDF1419. (2) Exome sequencing identified a predicted pathogenic variant of *MAP3K1* within the chromosome 5q interval. (3) Except for the p.(Arg1487His) variant of *MAP3K1*, the gDNA sequences of all annotated exons of the other six genes in this chromosome 5q 1.2 Mb interval are well covered in our exome data. There were no convincing predicted deleterious biallelic variants in the six other genes in this 1.2 Mb interval. (4) Two different biallelic variants of independently published *Map3k1* mouse models are deaf [5,6]. Optimistically, more families segregating hearing loss associated with additional recessive variants of *MAP3K1* (or singletons) will come to light following this report.

## Data Availability

The data presented (the variant of MAP3K1 segregating in family PKDF1419) in this study are openly available in Clinvar, at https://www.ncbi.nlm.nih.gov/clinvar/ (accessed on 20 February 2021), accession number SUB13512558.

## Supplementary Materials

The following supporting information can be downloaded at https://www.mdpi.com/article/10.3390/genes15070845/s1, Figure S1: Endocochlear potential (EP) measurement data of *Map3k1* mutant mouse; Figure S2: Expression of *Hgf-Met* signaling pathway genes amongst cochlear cell types; Figure S3: HGF-MET signaling pathway; Figure S4: Locations of our missense residue (bold red font) along with all reported missense variants of human *MAP3K1*. Table S1: (A) Additional variants identified after filtering exome data for individual LMG75-01, (B) additional variants on chromosome 5 that were identified after filtering exome data for individual LMG75-01, and (C) additional variants on chromosome 14 that were identified after filtering exome data for individual LMG75-01; Table S2: Variant classification using in silico tools and ACMG classification; Table S3: List of *MAP3K1* variants associated with reported phenotypes and their references; Table S4: Exome analyses of two affected individuals in two sibships (VI:3 and VI:8) from Family PKDF1419; Table S5: SpliceAI predicts an impact on splicing of NM_005921.2:c.4460G>A substitutions. Refs. [13,17,24,45,46,47,64] are cited in Supplementary Material.
