# Supplementary material for: Deafness DFNB128 Associated with a Recessive Variant of Human MAP3K1 Recapitulates Hearing Loss of Map3k1-Deficient Mice
Source: Genes (Basel). 2024 Jun 27;15(7):845. doi: 10.3390/genes15070845 (PMC11276321; doi:10.3390/genes15070845)
Supplement: Supplementary file 1 [file genes-15-00845-s001.zip › genes-3045527-supplementary.pdf]

Supplementary Information

Deafness DFNB128 associated with a recessive variant of human *MAP3K1* recapitulates hearing loss of *Map3k1* deficient mice

Rabia Faridi[1], Rizwan Yousaf[1,9], Sayaka Inagaki[1], Rafal Olszewski[2], Shoujun Gu[2], Robert J. Morell[3], Elizabeth Wilson[1], Ying Xia[4], Tanveer Ahmed Qaiser[5], Muhammad Rashid[6], Cristina Fenollar-Ferrer[1], Michael Hoa[2], Sheikh Riazuddin[7], Thomas B. Friedman[1]

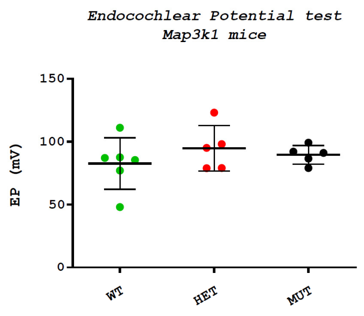

ANOVA summary	
F	0.7312
P value	0.5001
P value summary	ns
Significant diff. among means (P < 0.05)?	No
R squared	0.1011

Fig.S1. Endocochlear potential (EP) measurements of *Map3k1* mutant mouse. The method we used for EP measurements employed a glass micro-electrode that was inserted into the round window of the mouse as previously described in detail [1-3]. Anesthesia of mice used tribromoethanol (Avertin, 15.1 mg/ml) at 0.35 mg per gm body weight.

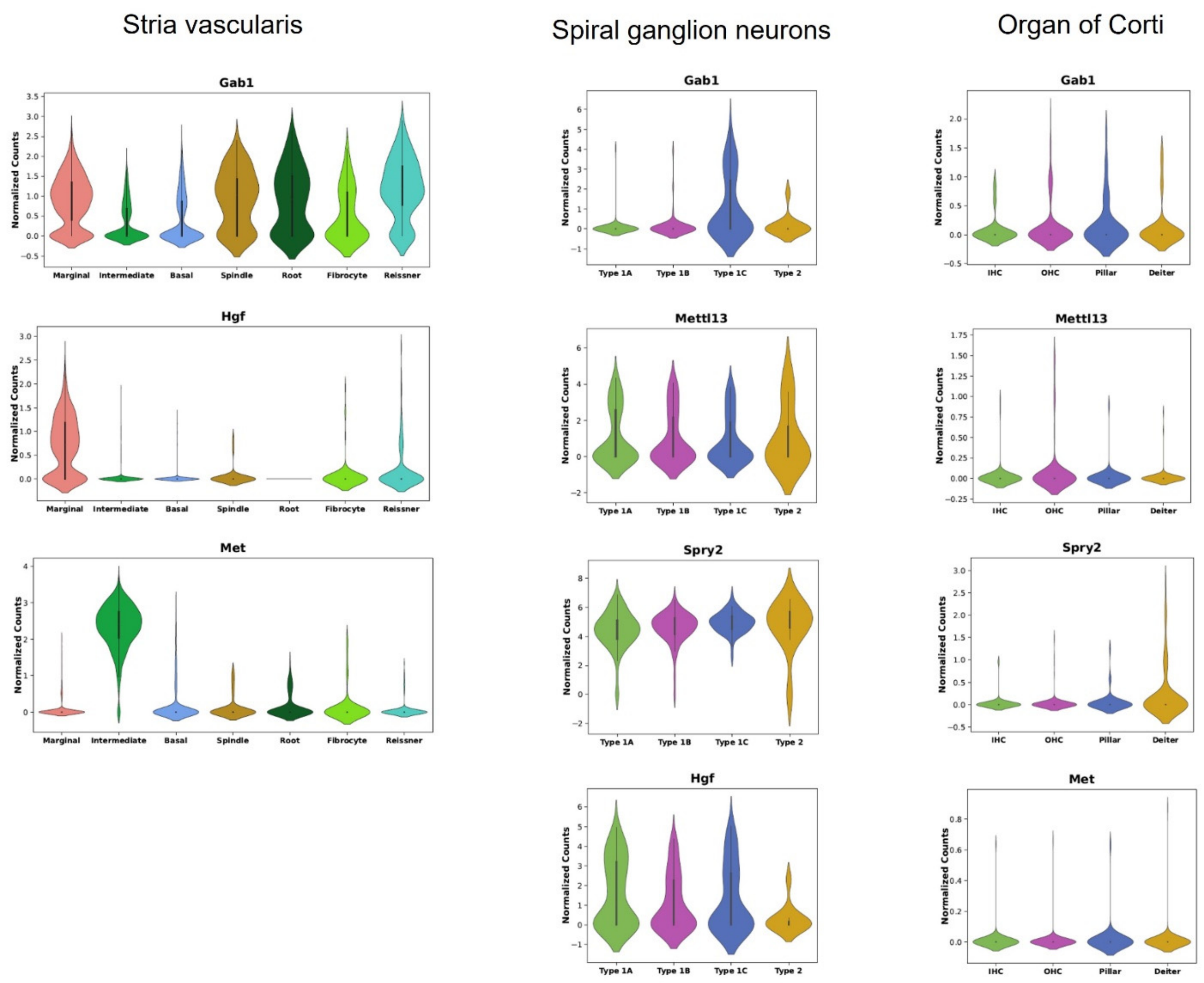

Fig. S2. Expression of *Hgf-Met* signaling pathway genes amongst cochlear cell types. First column on the left shows expression of *Hgf-Met* signaling pathway genes, including *Gab1*, *Hgf,* and *Met*, amongst stria vascularis cell types, including marginal, intermediate, basal and spindle cells as well as adjacent root cells, fibrocytes and Reissner's membrane. The middle column shows expression of *Hgf-Met* signaling pathway genes, including *Gab1*, *Mettl13*, *Spry2*, and *Hgf* amongst adult spiral ganglion neuron subtypes, including subtypes 1A, 1B, 1C and 2. The right most column shows expression of *Hgf-Met* signaling pathway genes, including *Gab1*, *Mettl13*, *Spry2*, and *Met* amongst P7 organ of Corti cell types, including inner hair cells (IHC), outer hair cells (OHC), pillar cells, and Deiters cells. Violin plots depict gene expression in normalized counts.

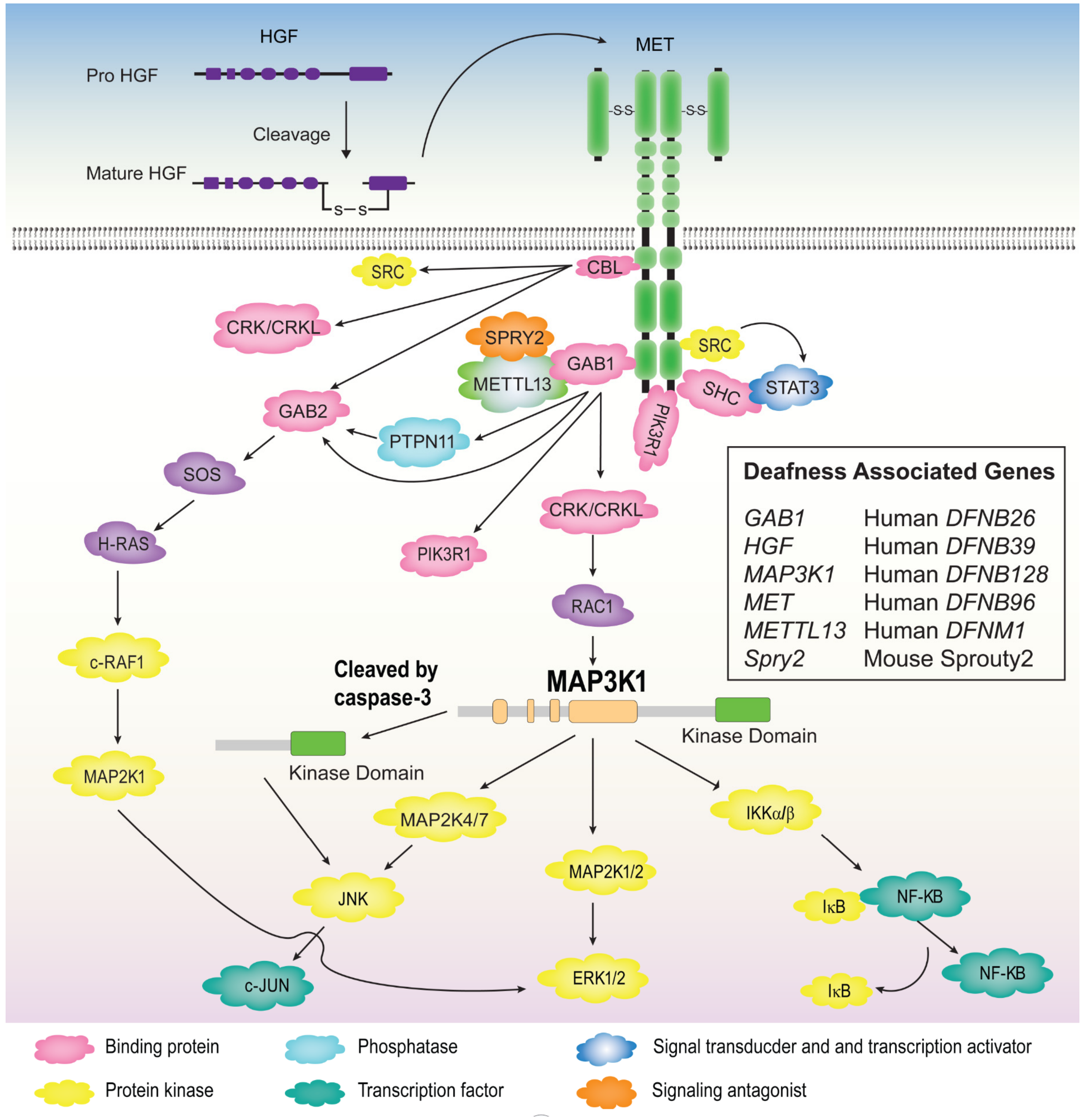

Fig. S3. HGF-MET signaling pathway

HGF-MET signaling pathway including MAP3K1 (Abridged). Mature HGF binds to MET and activates it through autophosphorylation. Binding proteins and direct kinase substrates activate downstream HGF-MET signaling pathway as shown. Full length MAP3K1 activate c-JUN, ERK18/2 and NF-κB pathways. MAP3K1 C-terminal fragment containing the kinase domain is formed by caspase-3 cleavage and activate JNK pathway. For further details, see references [3-7].

Abbreviations: CBL, Cbl proto-oncogene; c-JUN, Jun proto-oncogene, AP-1 transcription factor subunit; CRK, CRK proto-oncogene, adaptor protein; CRKL, CRK like proto-oncogene, adaptor protein; ERK1/2, Extracellular signal-regulated kinase 1 and 2; GAB1, GRB2 associated binding protein 1; GAB2, GRB2 associated binding protein 2; HGF, Hepatocyte growth factor; H-RAS, v-Ha-ras Harvey rat sarcoma viral oncogene homolog; IκB, IκB kinase; IKKα/β, IκB kinase alpha

and beta complex; JNK, c-Jun N-terminal kinase JNK; MAP2K1, mitogen-activated protein kinase kinase 1; MAP2K2, mitogen-activated protein kinase kinase 2; MAP2K4, mitogen-activated protein kinase kinase 4; MAP2K7, mitogen-activated protein kinase kinase 7; MAP3K1, mitogen-activated protein kinase kinase kinase 1; MET, MET proto-oncogene, receptor tyrosine kinase; METTL13, Methyltransferase 13, eEF1A lysine and N-terminal methyltransferase; NF-κB, Nuclear factor kappa-light-chain-enhancer of activated B cells; PIK3R1, phosphoinositide-3-kinase regulatory subunit 1; PTPN11, protein tyrosine phosphatase non-receptor type 11; RAC1, Rac family small GTPase 1 RAF1, Raf-1 proto-oncogene, serine/threonine kinase; SHC, SHC-adaptor protein SOS, Son of sevenless, SPRY2, Sprouty RTK signaling antagonist 2; SRC, SRC proto-oncogene, non-receptor tyrosine kinase; STAT3, Signal transducer and activator of transcription 3.

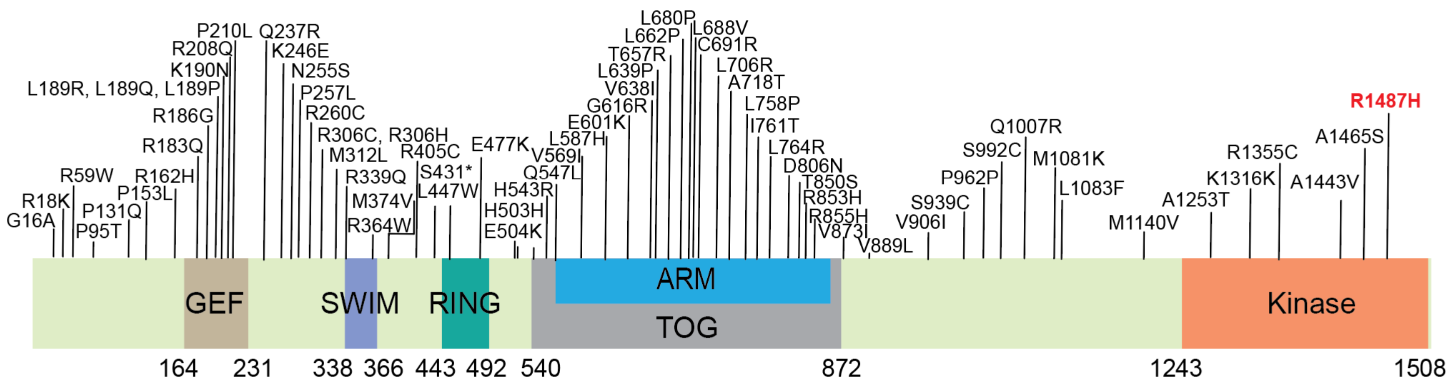

Figure S4: Locations of our missense residue (bold red font) along with all reported missense variants of human *MAP3K1*.

Table S1A. Additional variants identified after filtering exome data for individual LMG75-01.

Chr.	Position	End Position	Cytoband	Variation Type	Gene Symbol	Transcript ID	Protein Variant	Case Samples	Zygosity	Classification	CADD Score	gnomAD Frequency	Reason for exclusion	Other
1	116944819	116944819	p13.1	SNV	*PTGFRN*	NM_020440.4	p.V187M	LMG75_01	Heterozygous	VUS	22.3	0.016	High AF	
1	109479469	109479469	p13.3	SNV	*SYPL2*	NM_001040709.2	p.D247G	LMG75_01	Heterozygous	VUS	17.19	0.017	High AF	0.5421% gnomAD (South Asian)
1	110223777	110223777	p13.3	SNV	*KCNC4*	NM_004978.6	p.R498W	LMG75_01	Heterozygous	VUS	27.7	0.01	High AF	0.1656% gnomAD (South Asian)
1	100107664	100107664	p21.2	SNV	*SASS6*	NM_194292.3	p.E370D	LMG75_01	Heterozygous	VUS	25.3	0.006	High AF	0.1035% gnomAD (South Asian)
1	52033146	52033146	p32.3	SNV	*KTI12*	NM_138417.3	p.P206T	LMG75_01	Homozygous	VUS	20.3	0.021	High AF	
1	41037422	41037422	p34.2	SNV	*SCMH1*	NM_001350667.2	p.L530F	LMG75_01	Heterozygous	VUS	18.37	0.024	High AF	0.4965% gnomAD (South Asian)

1	43170863	43170863	p34.2	SNV	*EBNA1BP2*	NM_001195831.3	p.A169T	LMG75_01	Heterozygous	VUS	25.2	0.015	High AF	0.4758% gnomAD (South Asian)
1	32771398	32771398	p35.1	SNV	*KIAA1522*	NM_001369553.1;	p.P681L	LMG75_01	Heterozygous	VUS	23.6		High AF	
1	26197689	26197689	p36.11	SNV	*CATSPER4*	NM_198137.2	p.G155S	LMG75_01	Heterozygous	VUS	25.7	0.009	High AF	0.2068% gnomAD (South Asian)
1	21884821	21884821	p36.12	SNV	*HSPG2*	NM_005529.7	p.R485W	LMG75_01	Heterozygous	VUS	32	0.009	High AF	0.2069% gnomAD (South Asian)
1	21838914	21838914	p36.12	SNV	*HSPG2*	NM_005529.7	p.R3354H	LMG75_01	Heterozygous	VUS	16.25	0.003	High AF	0.0414% gnomAD (South Asian)
1	17308289	17308289	p36.13	SNV	*PADI4*	NM_012387.3	p.T23P	LMG75_01	Heterozygous	VUS	24.7	0.001	High AF	0.0414% gnomAD (South Asian)
1	10654079	10654079	p36.22	SNV	*CASZ1*	NM_001079843.3	p.V660M	LMG75_01	Heterozygous	VUS	20.5	0.018	High AF	0.4762% gnomAD (South Asian)
1	10640045	10640045	p36.22	SNV	*CASZ1*	NM_001079843.3	p.M1393L	LMG75_01	Heterozygous	VUS	22.3	0.016	High AF	0.4762% gnomAD (South Asian)
1	151763157	151763157	q21.3	SNV	*OAZ3*	NM_031420.4	p.G8R	LMG75_01	Heterozygous	VUS	15.24	0.026	High AF	0.5595% gnomAD (South Asian)
1	153681219	153681219	q21.3	SNV	*NPR1*	NM_000906.4	p.E321K	LMG75_01	Heterozygous	VUS	24.8	0.004	High AF	0.0207% gnomAD (South Asian)
1	165651033	165651033	q24.1	SNV	*MGST3*	NM_004528.4	p.T46M	LMG75_01	Heterozygous	VUS	24.7	0.019	High AF	
1	165654346	165654346	q24.1	SNV	*MGST3*	NM_004528.4	p.T106M	LMG75_01	Heterozygous	VUS	25.1	0.001	High AF	0.0207% gnomAD (South Asian)
1	169829325	169829325	q24.2	SNV	*C1orf112*	NM_001320047.2	p.P397L	LMG75_01	Heterozygous	VUS	26.9	0.001	High AF	0.0013% gnomAD (European)
1	180941116	180941116	q25.3	SNV	*KIAA1614*	NM_020950.2	p.S997N	LMG75_01	Heterozygous	VUS	12.41	0.013	High AF	0.3953% gnomAD (South Asian)
1	214645798	214645798	q41	SNV	*CENPF*	NM_016343.4	p.Q2076H	LMG75_01	Heterozygous	VUS	22.5	0.01	High AF	0.3103% gnomAD (South Asian)
1	223771896	223771896	q41	SNV	CAPN2	NM_001146068.2	p.R664Q	LMG75_01	Heterozygous	VUS	26	0.005	High AF	0.0169%

														gnomAD (African)
1	225358653	225358653	q42.12	SNV	DNAH14	NM_001367479.1	c.11776+1G>T	LMG75_01	Heterozygous	VUS	34	0.002	High AF	0.0621% gnomAD (South Asian)
1	226548954	226548954	q42.12	SNV	STUM	NM_001003665.4	p.V17G	LMG75_01	Heterozygous	VUS	12.59		No phenotypic association	
1	226548953	226548953	q42.12	SNV	STUM	NM_001003665.4	p.V17M	LMG75_01	Heterozygous	VUS	15.77		No phenotypic association	
1	228288069	228288069	q42.13	SNV	OBSCN	NM_052843.4	p.A3274T	LMG75_01	Heterozygous	VUS	15.22	0.014	High AF	0.3309% gnomAD (South Asian)
1	248593134	248593134	q44	SNV	OR2T10	NM_001004693.2	p.T212M	LMG75_01	Homozygous	VUS	12.28	0.007	High AF	0.0341% gnomAD (Latino)
LMG75_01 (PKDF1419:VI:3)	AF: Allele Frequency													

Table S1B. Additional variants on chromosome 5 that were identified after filtering exome data for individual LMG75-01.

Chromosome	Position	End Position	Cytoband	Variation Type	Gene Symbol	Transcript ID	Protein Variant	Case Samples	Zygosity	Classification	CADD Score	gnomAD Frequency	OMIM Phenotype
5	38530625	38530625	p13.1	Deletion	*LIFR*	NM_001364298.2	p.L8*	LMG75_01	Heterozygous	VUS	15.62	0.001	
5	56893601	56893601	q11.2	SNV	***MAP3K1***	NM_005921.2	p.R1487H	LMG75_01	Homozygous	VUS	31	0.001	
5	61531630	61531630	q12.1	SNV	*ZSWIM6*	NM_020928.2	p.R717Q	LMG75_01	Homozygous	VUS	31	0.02	Acromelic frontonasal dysostosis
5	67145327	67145327	q12.3	SNV	*MAST4*	NM_001393526.1	p.E1017D;	LMG75_01	Homozygous	VUS	13.39	0.002	Juvenile myoclonic epilepsy
5	73123453	73123453	q13.2	SNV	*TMEM171*	NM_001161342.3	p.V27A	LMG75_01	Homozygous	VUS	24.2	0.006	
5	95517049	95517049	q15	SNV	*TTC37*	NM_014639.4	p.R768H	LMG75_01	Heterozygous	VUS	20.7	0.014	
5	113513911	113513911	q22.2	SNV	*YTHDC2*	NM_001345976.2	p.S6C	LMG75_01	Heterozygous	VUS	25	0.033	
5	112878834	112878834	q22.2	SNV	*REEP5*	NM_005669.5	p.A174A; p.R118P	LMG75_01	Heterozygous	VUS	21.1	0.009	
5	115841521	115841521	q22.3	SNV	*ATG12*	NM_001284.4	p.L11P	LMG75_01	Heterozygous	VUS	24.5	0.005	
5	123399266	123399266	q23.2	SNV	*CEP120*	NM_153223.4	p.G135E; p.G161E	LMG75_01	Heterozygous	VUS	20.5	0.005	

5	136172512	136172512	q31.1	SNV	*SMAD5*	NM_001001419.3	p.E285G	LMG75_01	Heterozygous	VUS	29.8		
5	141394245	141394245	q31.3	SNV	*PCDHGA8*	NM_018912.3	p.D478N	LMG75_01	Heterozygous	VUS	31	0.197	
5	144473773	144473773	q31.3	SNV	*KCTD16*	NM_001370486.1	p.D316N	LMG75_01	Heterozygous	VUS	24.5	0.001	
5	140856227	140856227	q31.3	SNV	*PCDHA10*	NM_018911.3	p.Q60P	LMG75_01	Heterozygous	VUS	16.59	0.337	
5	141372812	141372812	q31.3	SNV	*PCDHGB3*	NM_018917.4	p.V806V	LMG75_01	Heterozygous	VUS	13.51	0.002	
5	145872919	145872919	q32	SNV	*GRXCR2*	NM_001080516.2	p.R17Q	LMG75_01	Heterozygous	Likely Pathogenic	23.8	0.003	
5	150650125	150650125	q33.1	SNV	*SYNPO*	NM_007286.6	p.S617W	LMG75_01	Heterozygous	VUS	19.91		
5	157106854	157106854	q33.3	SNV	*HAVCR2*	NM_032782.5	p.G56V	LMG75_01	Heterozygous	VUS	21.5		
5	177400351	177400351	q35.3	SNV	*PFN3*	NM_001029886.3	p.D76H	LMG75_01	Heterozygous	VUS	25.1	0.002	
5	179863693	179863693	q35.3	SNV	*TBC1D9B*	NM_198868.3;	p.L329V	LMG75_01	Heterozygous	VUS	17.08	0.003	
LMG75_01 (PKDF1419:VI:3)													

Table S1C. Additional variants on chromosome 14 that were identified after filtering exome data for individual LMG75-01.

Chromosome	Position	End Position	Cytoband	Variation Type	Gene Symbol	Transcript ID	Protein Variant	Case Samples	Zygosity	Classification	CADD Score	gnomAD Frequency	OMIM Phenotype	Other	
14	24187533	24187533	q12	SNV	*IPO4*	NR_051979.2	p.A152V	LMG75_01	Heterozygous	VUS	21.1	0.009			
14	44964106	44964106	q21.2	SNV	*TOGARAM1*	NM_001308120.2	p.G562V	LMG75_01	Heterozygous	VUS	24.8	0.012			
14	59465120	59465120	q23.1	SNV	*GPR135*	NM_022571.6	p.A36E	LMG75_01	Homozygous	VUS	19.54		No phenotypic association		
14	92071009	92071010	q32.12	Insertion	*ATXN3*	NM_001164778.2	p.G236delinsEQQQQR	LMG75_01	Heterozygous	VUS					
14	1.03E+08	1.03E+08	q32.32	SNV	*MARK3*	NM_001128921.3	p.R606G	LMG75_01	Heterozygous	VUS	25.5	0.001	Visual Impairment and progressive phthisis bulbi		
14	1.04E+08	1.04E+08	q32.33	SNV	*COA8*	NM_001302653.2	p.I126F	LMG75_01	Heterozygous	VUS	23.9	0.003			

14	1.04E+08	1.04E+08	q32.33	SNV	*COA8*	NM_001302653.2	p.E123K	LMG75_01	Heterozygous	VUS	23.8	0.013		0.0606% gnomAD (South Asian)	
14	1.04E+08	1.04E+08	q32.33	SNV	*KIF26A*	NM_015656.2	p.P953A	LMG75_01	Heterozygous	VUS	15.84	0.001			
LMG75_01 (PKDF1419:VI:3)															

Table S2. Variant classification using *in silico* tools and ACMG classification

	HGVS	gnomAD (v3.1.2) Max AF (%) (Max POP)	gnom AD v3.1.2 Homo	gnom AD v3.1.2 global AF (%)	gnom AD v2.1.1 Homo	gnomA D (v2.1.1) Max AF (%) (Max POP)	MetaRNN	FATHMM-MKL	CADD_ph red (v1. 6)	LRT	Mutation Assessor	Mutation Taster	REVEL
Family PKDF1419	c.4460G>A, p.(Arg1487His)	0	0	0.00066	0	0.001470 (EUR)	P	P	31	P	B	D	0.54

D= Deleterious

P= Pathogenic

B- Benign

EUR= European (non-Finnish)

Table S3. List of *MAP3K1* variants associated with reported phenotypes and their references

c.DNA	Protein	Reported phenotype	Reference
c.47G>C	Gly16Ala	Developmental disorder	Kaplanis (2020) Nature 586, 757<br>Zhou (2022) Nat Genet 54: 1305
c.53G>A	Arg18Lys	Developmental disorder	Kaplanis (2020) Nature 586, 757<br>Zhou (2022) Nat Genet 54: 1305
c.175C>T	Arg59Trp	Colorectal cancer	Fatemi (2023) Kaohsiung J Med Sci
c.283C>A	Pro95Thr	Breast cancer, male	Rizzolo et al (2019) Int J Cancer 145, 390
c.392C>A	Pro131Gln	46,XY DSD	Kalinchenko (2020) Probl Endokrinol
c.458C>T	Pro153Leu	DSD	Loke et al (2014) Hum Mol Genet 23, 1073, Chamberlin et al (2019) Hum Mol Genet 28: 1620
c.485G>A	Arg162His	Increased risk of neurodevelopmental disorder	Stessman et al (2017) Nat Genet 49, 515
c.548G>A	Arg183Gln	Hearing impairment	Schrauwen et al (2019) Eur J Hum Genet 27, 869
c.556A>G	Arg186Gly	46,XY DSD	Chen et al (2022) Front Genet 13,
c.566T>G	Leu189Arg	DSD	Pearlman et al (2010) Am J Hum Genet 87, 898, Eggers et al (2016) Genome Biol 17: 243, Chamberlin et al (2019) Hum Mol Genet 28: 1620, Loke et al (2012) Clin Genet 81: 272
c.566T>A	Leu189Gln	46,XY gonadal dysgenesis	Granados et al (2017) Am J Med Genet C Semin Med Genet 175, 253
c.566T>C	Leu189Pro	DSD	Pearlman et al (2010) Am J Hum Genet 87, 898, Chamberlin et al (2019) Hum Mol Genet 28: 1620, Loke et al (2012) Clin Genet 81: 272, Upadhyay et al (2018) Clin Genet 93: 412
c.570G>C	Lys190Asn	46,XY DSD	Yu et al (2022) Taiwan J Obstet Gynecol 61, 903
c.623G>A	Arg208Gln	46,XY DSD	Kalinchenko (2020) Probl Endokrinol
c.629C>T	Pro210Leu	46,XY DSD	Yu et al (2020) Asian J Androl 23, 69
c.710A>G	Gln237Arg	DSD	Mazen et al (2021) Am J Med Genet A 185, 1666
c.736A>G	Lys246Glu	DSD	Loke et al (2014) Hum Mol Genet 23, 1073, Chamberlin et al (2019) Hum Mol Genet 28: 1620
c.764A>G	Asn255Ser	Breast and/or gynecological cancer	Dominguez-Valentin et al (2018) Hered Cancer Clin Pract 16, 4
c.770C>T	Pro257Leu	DSD	Baxter et al (2015) J Clin Endocrinol Metab 100, E333, Capalbo et al (2019) PLoS Genet 15: e1008409
c.778C>T	Arg260Cys	46,XY DSD	Cheng et al (2020) Biosci Rep 40, BSR20200616, Stessman et al

			(2017) Nat Genet 49: 515, Cheng et al (2020) Biosci Rep 40
c.916C>T	Arg306Cys	Sepsis, modifier of	Taudien et al (2016) EBioMedicine 12, 227
c.917G>A	Arg306His	46,XY DSD	Tsai (2023) Biomedicines 11, 242
c.934A>T	Met312Leu	46,XY DSD	Eggers et al (2016) Genome Biol 17, 243
c.1016G>A	Arg339Gln	DSD	Baxter et al (2015) J Clin Endocrinol Metab 100, E333
c.1090C>T	Arg364Trp	Congenital diaphragmatic hernia	Qiao (2021) Am J Hum Genet 108, 1964
c.1120A>G	Met374Val	46,XY DSD	Kalinchenko (2020) Probl Endokrinol
c.1213C>T	Arg405Cys	Developmental disorder	Turner et al (2019) Am J Hum Genet 105, 1274
c.1292C>G	Ser431Ter	Developmental disorder	Kaplanis (2020) Nature 586, 757<br>Zhou (2022) Nat Genet 54: 1305
c.1340T>G	Leu447Trp	46,XY DSD	Machado et al (2017) Arch Endocrinol Metab 61 S48
c.1429G>A	Glu477Lys	MAP3K1-related disorder	Maron (2023) JAMA 330, 161
c.1509C>T	His503His	Autism spectrum disorder	Fu (2022) Nat Genet 54, 1320<br>Zhou (2022) Nat Genet 54: 1305
c.1510G>A	Glu504Lys	46,XY DSD	Kalinchenko (2020) Probl Endokrinol
c.1628A>G	His543Arg	46,XY DSD	Gomes (2022) J Clin Endocrinol Metab 107
c.1640A>T	Gln547Leu	Breast cancer	Xie et al (2018) Clin Genet 93, 41
c.1705G>A	Val569Ile	Breast cancer	Liu et al (2017) Cancer Med 6, 547
c.1760T>A	Leu587His	46,XY gonadal dysgenesis	Granados et al (2017) Am J Med Genet C Semin Med Genet 175, 253
c.1801G>A	Glu601Lys	Breast cancer	Xie et al (2018) Clin Genet 93, 41
c.1846G>A	Gly616Arg	DSD	Pearlman et al (2010) Am J Hum Genet 87, 898, Capalbo et al (2019) PLoS Genet 15: e1008409, Chamberlin et al (2019) Hum Mol Genet 28: 1620
c.1912G>A	Val638Ile	Hypercholesterolemia	Marmontel et al (2020) Clin Genet 98, 589, Gomes et al (2018) Clin Endocrinol (Oxf) 89: 164,
c.1916T>C	Leu639Pro	46,XY DSD	Machado et al (2017) Arch Endocrinol Metab 61 S48
c.1970C>G	Thr657Arg	46,XY DSD	Machado et al (2017) Arch Endocrinol Metab 61 S48, Al Shamsi et al (2020) Mol Genet Genomic Med 8: e1514
c.1985T>C	Leu662Pro	46,XY DSD	Yu et al (2020) Asian J Androl 23, 69
c.2039T>C	Leu680Pro	46,XY DSD	Kalinchenko (2020) Probl Endokrinol
c.2062C>G	Leu688Val	Gonadal dysgenesis	Xu (2019) Eur J Endocrinol 181, 311<br>Tang (2023) Endocr Connect
c.2071T>C	Cys691Arg	46,XY gonadal dysgenesis	Eggers et al (2016) Genome Biol 17, 243, Machado et al (2017) Arch Endocrinol Metab 61: S48

c.2117T>G	Leu706Arg	46, XY gonadal dysgenesis	Xue et al (2019) Gene 718, 144072
c.2152G>A	Ala718Thr	Increased risk of neurodevelopmental disorder	Stessman et al (2017) Nat Genet 49, 515
c.2273T>C	Leu758Pro	DSD	Stranneheim et al (2021) Genome Med 13, 40
c.2282T>C	Ile761Thr	46,XY DSD	Cheng et al (2021) Orphanet J Rare Dis 16, 268
c.2291T>G	Leu764Arg	46,XY gonadal dysgenesis	Granados et al (2017) Am J Med Genet C Semin Med Genet 175, 253
c.2416G>A	Asp806Asn	Asthma, association with	Szczepankiewicz et al (2012) J Asthma 49, 329, Das et al (2013) Indian J Hum Genet 19: 437
c.2549C>G	Thr850Ser	Developmental disorder	Kaplanis (2020) Nature 586, 757<br>Zhou (2022) Nat Genet
c.2558G>A	Arg853His	Increased risk of neurodevelopmental disorder	Stessman et al (2017) Nat Genet 49, 515
c.2564G>A	Arg855His	Increased risk of neurodevelopmental disorder	Stessman et al (2017) Nat Genet 49, 515
c.2617G>A	Val873Ile	Breast cancer	Liu et al (2017) Cancer Med 6, 547, Xie et al (2018) Clin Genet 93: 41
c.2665G>C	Val889Leu	Breast cancer	Xie et al (2018) Clin Genet 93, 41
c.2716G>A	Val906Ile	Acute respiratory distress syndrome, association with	Morrell et al (2018) Am J Respir Cell Mol Biol 58, 117
c.2816C>G	Ser939Cys	Breast and/or gynecological cancer	Dominguez-Valentin et al (2018) Hered Cancer Clin Pract 16, 4
c.2886C>G	Pro962Pro	Developmental disorder	Kaplanis (2020) Nature 586, 757<br>Zhou (2022) Nat Genet
c.2975C>G	Ser992Cys	Breast cancer, male	Rizzolo et al (2019) Int J Cancer 145, 390
c.3020A>G	Gln1007Arg	46,XY DSD	Cheng et al (2022) Mol Med Rep 26,
c.3242T>A	Met1081Lys	46,XY DSD	Kalinchenko (2020) Probl Endokrinol
c.3247C>T	Leu1083Phe	Breast cancer, male	Rizzolo et al (2019) Int J Cancer 145, 390
c.3418A>G	Met1140Val	46,XY DSD	Tsai (2023) Biomedicines 11,
c.3757G>A	Ala1253Thr	Breast cancer	Xie et al (2018) Clin Genet 93, 41
c.3948G>A	Lys1316Lys	Developmental disorder	Kaplanis (2020) Nature 586, 757<br>Zhou (2022) Nat Genet
c.4063C>T	Arg1355Cys	Increased risk of neurodevelopmental disorder	Stessman et al (2017) Nat Genet 49, 515
c.4328C>T	Ala1443Val	46,XY DSD	Eggers et al (2016) Genome Biol 17, 243
c.4393G>T	Ala1465Ser	Autism	Zhou (2022) Nat Genet 54, 1305
c.634-8T>A		DSD	Pearlman et al (2010) Am J Hum Genet 87, 898, Chamberlin et al (2019) Hum

			Mol Genet 28: 1620, Loke et al (2012) Clin Genet 81: 272
c.1035+1G>T		Increased risk of neurodevelopmental disorder	Stessman et al (2017) Nat Genet 49, 515
c.2180-2A>G		DSD	Chamberlin et al (2019) Hum Mol Genet 28: 1620, Loke et al (2012) Clin Genet 81: 272
c.2254C>T		46,XY DSD	Igarashi et al (2020) Sci Rep 10, 17375
c.-79579C>A		Reduced risk of breast cancer	Glubb et al (2015) Am J Hum Genet 96, 5
c.-78308T>C		Associated with risk of breast cancer	Glubb et al (2015) Am J Hum Genet 96, 5
c.-58706T>C		Increased risk of breast cancer	Glubb et al (2015) Am J Hum Genet 96, 5
c.-57922C>T		Increased risk of breast cancer	Glubb et al (2015) Am J Hum Genet 96, 5
c.483-18151A>G		Increased risk of breast cancer	Glubb et al (2015) Am J Hum Genet 96, 5
c.2073_2076delTGCA		Autism	Zhou (2022) Nat Genet 54, 1305
c.2845_2847delACA	p.(Thr949del)	Pancreatic cancer	Ma et al (2020) Cancer Biomark 27, 389
c.3111delA	p.(Asp1038Thrfs*44)	Breast cancer	Aloraifi et al (2015) FEBS J 282, 3424
c.14_16dupCGG	p.(Ala5dup)	46,XY DSD	Granados et al (2017) Am J Med Genet C Semin Med Genet 175, 253
c.1369dupA	p.(Thr457Asnfs*4)	Increased risk of neurodevelopmental disorder	Stessman et al (2017) Nat Genet 49, 515
c.4151dupT	p.(Arg1385Lysfs*35)	Breast cancer	Lhota et al (2016) Clin Genet 90, 324, Stessman et al (2017) Nat Genet 49, 515
c.4531dupA	p.(Thr1511Asnfs*12)	Congenital heart disease	Morton et al (2021) JAMA Cardiol 6, 457
c.823_824delTCinsCT	p.(Ser275Leu)	Increased risk of neurodevelopmental disorder	Stessman et al (2017) Nat Genet 49, 515
DSD = Disorders of Sex Development			

Table S4: Exome analyses of two affected individuals in two sibships (VI:3 and VI:8) from Family PKDF1419

As separate excel file.

Table S5. SpliceAI predicts an impact on splicing of NM_005921.2:c.4460G>A substitutions.

Genes	Variants	Type of mutations	SpliceAI predictions*			
			Acceptor Loss	Donor Loss	Acceptor Gain	Donor Gain
MAP3K1	NM_005921.2:c.4460G>A	Arg1487His	0.02	0	0	0
MAP3K1	NM_005921.2:c.4461T>C	Arg1487Arg<br>Silent mutation<br>(Negative control)	0.02	0	0.01	0
LRP2	NM_004525.3:c.7715 + 3A > T	p.(Gln2573LeufsTer11)<br>Splice-altering variant<br>(Positive control)	0.07	0.79	0	0.59

*SpliceAI score, a probability of the variant which may affect splicing.
Values with < 0.2 have a low probability of causing an abnormal splice.
Values 0.2 to 0.5 have a predicted splice abnormality that is uncertain, and a value > 0.8 predicts that the variant is likely to cause an abnormal splice event.

References

1. Wangemann, P.; Itza, E.M.; Albrecht, B.; Wu, T.; Jabba, S.V.; Maganti, R.J.; Lee, J.H.; Everett, L.A.; Wall, S.M.; Royaux, I.E.; et al. Loss of KCNJ10 protein expression abolishes endocochlear potential and causes deafness in Pendred syndrome mouse model. *BMC Med* **2004**, *2*, 30, doi:10.1186/1741-7015-2-30.
2. Wangemann, P.; Nakaya, K.; Wu, T.; Maganti, R.J.; Itza, E.M.; Sanneman, J.D.; Harbidge, D.G.; Billings, S.; Marcus, D.C. Loss of cochlear HCO3- secretion causes deafness via endolymphatic acidification and inhibition of Ca2+ reabsorption in a Pendred syndrome mouse model. *Am J Physiol Renal Physiol* **2007**, *292*, F1345-1353, doi:10.1152/ajprenal.00487.2006.
3. Morell, R.J.; Olszewski, R.; Tona, R.; Leitess, S.; Wafa, T.T.; Taukulis, I.; Schultz, J.M.; Thomason, E.J.; Richards, K.; Whitley, B.N.; et al. Noncoding Microdeletion in Mouse Hgf Disrupts Neural Crest Migration into the Stria Vascularis, Reduces the Endocochlear Potential, and Suggests the Neuropathology for Human Nonsyndromic Deafness DFNB39. *J Neurosci* **2020**, *40*, 2976-2992, doi:10.1523/JNEUROSCI.2278-19.2020.
4. Chamberlin, A.; Huether, R.; Machado, A.Z.; Groden, M.; Liu, H.M.; Upadhyay, K.; O, V.; Gomes, N.L.; Lerario, A.M.; Nishi, M.Y.; et al. Mutations in MAP3K1 that cause 46,XY disorders of sex development disrupt distinct structural domains in the protein. *Hum Mol Genet* **2019**, *28*, 1620-1628, doi:10.1093/hmg/ddz002.
5. Pham, T.T.; Angus, S.P.; Johnson, G.L. MAP3K1: Genomic Alterations in Cancer and Function in Promoting Cell Survival or Apoptosis. *Genes Cancer* **2013**, *4*, 419-426, doi:10.1177/1947601913513950.
6. Zhang, L.; Wang, W.; Hayashi, Y.; Jester, J.V.; Birk, D.E.; Gao, M.; Liu, C.Y.; Kao, W.W.; Karin, M.; Xia, Y. A role for MEK kinase 1 in TGF-beta/activin-induced epithelium movement and embryonic eyelid closure. *EMBO J* **2003**, *22*, 4443-4454, doi:10.1093/emboj/cdg440.
7. Grotegut, S.; von Schweinitz, D.; Christofori, G.; Lehembre, F. Hepatocyte growth factor induces cell scattering through MAPK/Egr-1-mediated upregulation of Snail. *EMBO J* **2006**, *25*, 3534-3545, doi:10.1038/sj.emboj.7601213.
